# Role of Diphosphonates Bone Scintigraphy in Correlation with Biomarkers for a Personalized Approach to ATTR Cardiac Amyloidosis in North-Eastern Romania

**DOI:** 10.3390/diagnostics13010083

**Published:** 2022-12-28

**Authors:** Teodor Marian Ionescu, Manuela Ciocoiu, Raoul-Vasile Lupușoru, Irena Grierosu, Radu Andy Sascău, Wael Jalloul, Roxana Iacob, Cati Raluca Stolniceanu, Alexandra Clement, Ana-Maria Stătescu, Daniela Crișu, Antoniu Octavian Petriș, Florin Mitu, Cipriana Ștefănescu

**Affiliations:** 1 Department of Biophysics and Medical Physics, University of Medicine and Pharmacy “Grigore T. Popa” Iasi, 700115 Iasi, Romania; 2 Department of Pathophysiology, University of Medicine and Pharmacy “Grigore T. Popa” Iasi, 700115 Iasi, Romania; 3 Nuclear Medicine Laboratory, “Sf. Spiridon” Emergency County Clinical Hospital Iasi, 700111 Iasi, Romania; 4 Cardiology Department, Cardiovascular Diseases Institute “Prof. Dr. George I.M. Georgescu”, 700503 Iasi, Romania; 5 Department of Internal Medicine-Cardiology, University of Medicine and Pharmacy “Grigore T. Popa” Iasi, 700115 Iasi, Romania; 6 Nuclear Medicine Laboratory, Regional Institute of Oncology Iasi, 700483 Iasi, Romania; 7 Cardiology Department, “Sf. Spiridon” Emergency County Clinical Hospital Iasi, 700111 Iasi, Romania; 8 Cardiology Department, Clinical Recovery Hospital Iasi, 700661 Iasi, Romania

**Keywords:** ATTR, bone scintigraphy, biomarkers

## Abstract

Transthyretin cardiac amyloidosis (ATTR) is a rare cardiac protein deposition disease characterized by progressive thickening of both ventricles, the inter-atrial-ventricular septum and the atrioventricular valves. The gold standard method for diagnosing this rare pathology is endomyocardial biopsy. If this method cannot be used, the alternative is a mixture of clinical and paraclinical tests. Over the course of five years, we examined 58 patients suspected of cardiac amyloidosis based on electrocardiography and ultrasonography criteria, who had been sent for bone scintigraphy in order to determine the presence of ATTR cardiac amyloidosis. However, the final diagnosis was set by correlating the bone scan with genetic testing, free light chain dosage or soft tissue biopsy. Based on the final diagnosis we analyzed the patients’ predominant biomarkers in order to determine a possible correlation between them. This analysis is designed to help the general practitioner set a possible cardiac amyloidosis diagnosis.

## 1. Introduction

Amyloidosis is a rare protein deposition disease characterized by the buildup of amyloid in different organs and tissues affecting their normal function and leading to organ failure. According to classifications in literature, it may be primary or secondary, localized or systemic. Specifically, cardiac amyloidosis may be either localized or systemic. Based on their etiology, the proteins that determine this pathology are light chain amyloidosis (AL), transthyretin amyloidosis (ATTR), serum amyloid A amyloidosis (AA), apolyprotein A amyloidosis (AApo AI/II), fibrinogen amyloidosis (AFib) and gelsolin amyloidosis (AGel) [1,2,3].

According to the European Society of Cardiology’s (ESC) 2021 guidelines, the gold standard method for detecting cardiac amyloidosis is endomyocardial biopsy. However, due to the disadvantages of this method (its inability to diagnose the subtype involved, its low accessibility and its highly invasive nature), alternative diagnostic algorithms have been tested [3,4,5]. When cardiac amyloidosis is suspected (more than 12 mm thickening of the left ventricle wall in a patient over 65 years of age, in addition to at least one “red flag” criterion), the guidelines recommend simultaneous bone scan examination and free light chain dosage (FLC) [6]. The so-called “red flags” guide the physicians’ diagnostic algorithm towards a possible cardiac amyloidosis: patients over 65 years of age that may suffer from heart failure, aortic stenosis, normotensive or hypotensive but with a history of hypertension, autonomous dysfunction and sensory-motor impairment, peripheral polyneuropathy, proteinuria, ecchymosis, bilateral carpal tunnel syndrome, bicep muscle tendon rupture, transmural or subendocardic LGE or increased extracellular volume, reduced longitudinal strain with apical sparing, microvoltage, pseudoinfarct pattern, atrioventricular conduction disorders and family history [3,5,7].

The most common types of cardiac amyloidosis are ATTR and AL cardiac amyloidosis. Cardiological criteria for detecting ATTR or AL cardiac amyloidosis have been studied in the literature. They rely on biomarkers, electrocardiography (ECG) and ultrasonography criteria.

Electrocardiography (ECG) is the first step in guiding the diagnostic algorithm towards a possible cardiac amyloidosis. The criteria described in literature are based on low voltage (due to increased thickness of the left ventricle), Sokolow criteria, pseudo-infarct pattern, conduction disorders (complete or incomplete bundle branch blocks) and rhythm disorders (arrhythmias, atrial fibrillation, flutter) [8,9,10,11,12]. However, these criteria are found in both ATTR and AL cardiac amyloidosis but in different positivity rates [2,3,13].

Ultrasonography constitutes the second step in the diagnostic process. Although it cannot accurately determine which form of cardiac amyloidosis is involved, this method may help set the general diagnosis of cardiac amyloidosis. The main cardiac amyloidosis criteria are wall thickness (mean wall thickness for AL—15 mm ± 2; ATTR—17 mm ± 2), left ventricle ejection fraction (diastolic dysfunction in the early stages which is considered an important finding; systolic dysfunction occurs in later stages) and strain technique (depressed longitudinal strain in the basal and midventricular sections but preserved in the apical one; reduced global longitudinal strain, with apical sparing providing a specific appearance of “cherry on top” or “cupcake”) [2,3,8,9,12,14,15,16,17,18,19].

The third step is bone scintigraphy, which is a non-invasive method that studies the biodistribution of bisphosphonates at the skeletal level. This method is usually recommended in various neoplastic and non-neoplastic diseases. According to literature and the ESC guidelines, it is also highly specific for ATTR cardiac amyloidosis [20]. The standard uptake mechanism for bone scintigraphy is based on the absorption (of the radiotracer molecules) to the surface of hydroxyapatite crystals, depending on local bone vascularization and osteoblastic activity [21,22]. However, the exact uptake mechanism for cardiac amyloidosis remains unknown. There are several theories explaining cardiac uptake such as high calcium levels or microcalcification due to old age and long disease progression. It may also depend on the duration of amyloid deposition in ATTR compared to AL or on fibril accumulation over time. The uptake mechanism also depends on the radiopharmaceutical agent used. According to literature, the sensitivity and specificity differ from one radiotracer to another. 2,3-dicarboxypropane-1,1-diphosphonate (DPD) and pyrophosphate (PYP) exhibit the highest sensitivity and specificity for detecting cardiac amyloidosis. However, there are several papers stating that hydroxymethylene diphosphonate (HMDP)/hydroxyethylene diphosphonate (HDP) showed similar results to DPD for detecting this type of disease. The lowest sensitivity and specificity were shown by methylene diphosphonate (MDP) [23,24,25]. The acquired bone scan images are evaluated using the Perugini visual score and associating it with ratios specific to various regions of interest (ROI) [26,27]. According to the American Society of Nuclear Cardiology (ASNC) and European Association of Nuclear Medicine (EANM), a visual score of 2 or 3 was suggestive of ATTR cardiac amyloidosis, 0 ruled out this diagnosis, while 1 was considered equivocal. Myocardium uptake was also compared to rib uptake in order to determine whether the uptake was higher or not [28,29,30].

Depending on the bone scan result, the next step is either genetic testing (for a visual score of 2 or 3; it is able to determine the ATTR subtype involved) or a free light chain dosage, or FLC (for a visual score of 1 or 2; it has demonstrated high specificity for AL) [31,32,33].

However, the first step in a cardiological diagnostic algorithm is usually represented by blood tests or biomarkers. Therefore, a correlation between the bone scan results and the main type of biomarkers used in cardiology assessments may reveal a possible correlation. NT-proBNP and troponin T are the main biomarkers used to detect this pathology. According to literature, NT-proBNP has shown lower levels in ATTR than in AL. They were also considered possible predictors of mortality in cardiac amyloidosis [2,34]. Another prognostic classification was made by Chacko et al. who analyzed the estimated glomerular filtration rate (eGFR) and concluded that ATTR may have three stages: stage 1 (NT-proBNP and troponin T levels are below the threshold), stage 2 (either value is above the threshold) and stage 3 (both NT-proBNP and troponin T levels are above the threshold) [35]. Another classification was made by Gilmore et al. based on the correlation between NT-proBNP and eGFR values in order to stratify the ATTR subtypes into prognostic categories. The patients in this paper were also included in any of stages 1 to 3, based on their NT-proBNP and eGFR values [36]. The correlation between these biomarkers and the bone scan findings may be a future step in a more accurate diagnosis of cardiac amyloidosis (ATTR and AL).

## 2. Materials and Method

The patients were selected based on the following criteria: patients with heart failure of unknown cause, patients suspected of cardiac amyloidosis from a cardiological point of view, first degree relatives of patients diagnosed with cardiac amyloidosis or that died at a young age being apparently healthy.

The patients that refused to participate in the study and pregnant patients to whom bone scan was contraindicated were excluded from the study.

58 patients suspected of cardiac amyloidosis that were referred to the “Valeriu Rusu” Nuclear Medicine Laboratory of “Sf. Spiridon” Clinical Emergency Hospital of Iasi County were examined over the course of 5 years.

All the patients signed an informed consent which explained the procedure that they were going to undertake and that the investigation was not conditioned by their participation in the study. The consent also stated that, in case of its publication, their identity will be protected in accordance with the General Data Protection Regulation applying in the European Union.

The protocols implemented during the study were approved by the Ethics Committee of both the “Grigore T. Popa” University of Medicine and Pharmacy Iasi, Romania and “Sf. Spiridon” Clinical Emergency Hospital of Iasi County and therefore presented in the informed consent signed by the patient.

The diagnostic algorithm used when cardiac amyloidosis was suspected was based on literature and the ESC guidelines for diagnosing patients with possible cardiac amyloidosis. To that end all the patients underwent the standard diagnostic algorithm in case of heart failure of unknown cause. The algorithm included clinical and paraclinical investigation that were meant to direct the physician toward a possible diagnosis of cardiac amyloidosis. Standard clinical and paraclinical investigation included: the patients’ symptoms (dyspnea, fatigue, possible carpal tunnel syndrome and so on), personal and family medical history, biomarkers (NT-proBNP, urea, creatinine), ECG and ultrasonography. If these investigations raised a suspicion of cardiac amyloidosis, the patient was referred to the Nuclear Medicine Laboratory for a bone scintigraphy in order to confirm or rule out ATTR cardiac amyloidosis.

The administered radiotracer dosage was calculated based in accordance with the EANM guidelines for standard bone scintigraphy. The patients underwent ^99m^Tc-HDP bone scintigraphy using a Siemens Dual-Head Gamma Camera (Dual-Head Variable Angle Cardio Siemens e.cam Gamma Camera from Siemens Medical Solutions Inc. USA 2007) equipped with low energy high resolution planar collimators.

The acquisition protocol for cardiac amyloidosis included early images (whole body/static centered-on-the-thorax), followed by delayed images after 3 h (whole body, static and Single Photon Emission Computed Tomography—SPECT—centered-on-the-thorax).

Images were processed and interpreted by visual scoring (Perugini score), in addition to a personalized semiquantitative method. The semiquantitative method was based on drawing regions of interest (ROI) over the myocardium on static and SPECT centered on the thorax images. On the static images, the ROI of the heart did not include the ribs and the mirror tool used to maintain the size of the original ROI enabled us to obtain 2 additional ROI that were placed on the rib and on the contralateral side between the ribs. Ratios were obtained by comparing the uptake of the heart to that of the rib and the contralateral side. On the SPECT images the heart ROI was mirrored to the contralateral side on coronal and transverse incidence. Ratios were obtained by comparing the uptake of the heart with that of the contralateral side on both incidences. The results of the semiquantitative method were classified in accordance with the table below (Table 1).

The ranges for the semiquantitative ratio results were obtained by averaging the results of the ratios for all the patients. Then, that average was compared with the average result for each category of patients: ATTR, AL and non-ATTR.

Depending on the result of the bone scan, the patients were recommended to undergo either genetic testing and/or FLC dosage. The genetic test was based on gene sequencing by NGS short read Illumina, with an accuracy of 99.8% for the identification of point mutations. The test results were obtained either from the patients on the next follow-up session (for genetic testing) or from the hospital laboratory (for FLC dosage).

The results of the bone scan were reverse engineered and correlated with the patients’ biomarkers. For a more accurate correlation, the patients were initially classified according to the bone scan result into ATTR positive and possible AL. However, by relying on the genetic and FLC results, the classification was restructured as being ATTR positive, AL positive and non-ATTR. The investigation was focused especially on the ATTR and AL groups of patients.

Based on this classification, the modifications of each biomarker the patients were tested for were studied in order to determine a possible correlation. The results were also statistically analyzed and correlated by using the statistical analysis program (SPSS).

## 3. Results

### 3.1. ATTR Patients

The bone scan showed high myocardium uptake in 11 of the 58 patients in the group. These patients were ATTR positive. The diagnosis was set by correlating the visual score with the semiquantitative method.

**Figure 1:**
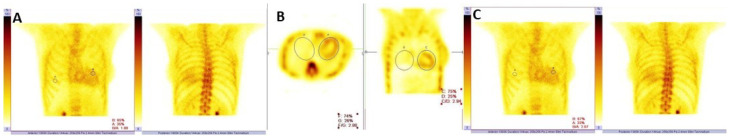
46 year old female patient with 3 on the bone scan a visual score, H/Rib = 1.88 (image (**A**)), H/Cl region = 2.94 and 2.80 on SPECT images (image (**B**)) followed by 2.07 on static images (image (**C**)); genetic testing revealed the presence of ATTRm with Glu74Gln genetic mutation; the eGFR value (calculated by MDRD and Cockcroft-Gault formula) determined stage 1 kidney disease. Images obtained from the archives of the Nuclear Medicine Laboratory of “Sf. Spiridon” Clinical Emergency Hospital of Iași County.

The Perugini visual score for these patients showed high uptake, with 4 patients scoring 2 and 7 patients scoring 3 (Figure 1).

The static centered-on-the-thorax-image-ratios confirmed the visual score with results ranging from 0.91 to 2.08 on the heart to rib ratio and from 1.2 to 2.71 on the heart to contralateral region ratio. The patient number from the original lot was maintained to avoid confusion (Table 2).

The heart-to-contralateral ratio calculated on the SPECT images also confirmed the visual score in all of the ATTR positive patients with results ranging from 1.12 to 2.97 on transverse incidence and from 0.98 to 2.98 on coronal incidence (Table 3).

Comparison between the static image ratios and the SPECT image ratios revealed results higher than the lowest value shown in Table 1 (Table 4) in almost all the patients in the group.

Only 6 of the 11 ATTR positive patients underwent genetic testing. Sequencing analysis for exons 1, 2, 3 and 4 showed pathogenic variants: c.220G > C (p.E74Q) or c.424G > A (p.V142I) in heterozygous status in the TTR gene. The reference sequence was TTR: NM_000371.3. Therefore, the genetic test confirmed the bone scan result and classified the patients as ATTRwt and ATTRm. The genetic mutation involved in ATTRm was determined. Based on the results of the genetic test, the patients’ background was further investigated, namely their family history. However, most of them could not specify if there was any kind of family history of heart conditions or, if they were aware of such conditions, they did not know the actual diagnoses. To that end, the patients were classified depending on the presence or absence of heart disease in the family (Table 5).

The biomarkers dosed for the ATTR patients were as follows: NT-proBNP, urea and creatinine. Based on these biomarkers, a goal was to extrapolate by what an ATTR positive patient would be characterized.

NT-proBNP was dosed in only 8 of the 11 ATTR positive patients and ranged between 1379 and 10,602 pg/mL. For a better appreciation of the exact range, the results were divided in groups of 1000 pg/mL. Subsequently, it was noted that most of the patients showed values either between 1001 and 2000 pg/mL or extreme values (>10,001 pg/mL) (Table 6).

**Figure 2:**
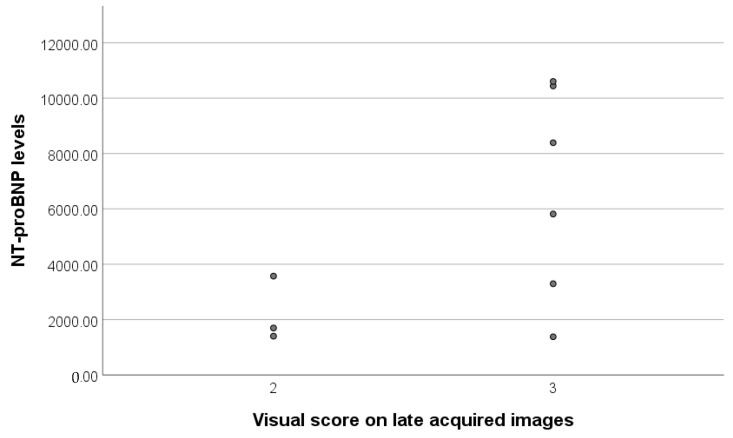
NT-proBNP levels in relation to the Visual Score results (Perugini score).

Correlation between the NT-proBNP levels of ATTR positive patients showed that most patients with the visual score 3 had extreme values of this biomarker (Figure 2).

**Figure 3:**
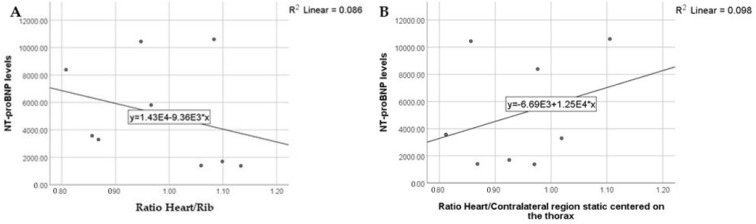
NT-proBNP levels in relation to the Heart/Rib (**A**) and Heart/Contralateral region (static centered-on-the-thorax images) (**B**) ratio results.

**Figure 4:**
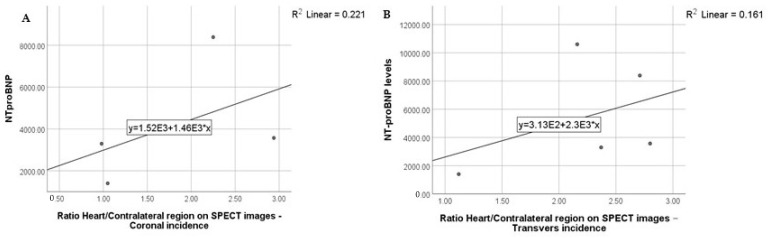
NT-proBNP levels in relation to the Heart/Contralateral region on SPECT images (Coronal incidence—(**A**); Transvers incidence—(**B**)) ratio results.

The correlation between the semiquantitative method and the NT-proBNP levels revealed the following:

- A: heart/rib ratio; the highest NT-proBNP levels were detected in patients with ratios between 0.9 and 1.1; the NT-proBNP levels were also high when the ratios were between 1.1 and 1.2 but did not have extreme values;
- B: heart/contralateral-region-on-static-images ratio; the highest NT-proBNP levels were detected in patients with ratios between 0.8 and 1.2; the NT-proBNP levels were also high when the ratios were between 0.8 and 1.1, but did not have extreme values (Figure 3)

- Heart/contralateral region ratio on SPECT images: the highest NT-proBNP levels were detected in patients with ratios between 2 and 3 (Figure 4)

Urea and creatinine were dosed for almost all of the ATTR patients. Urea was dosed for 8 patients and elevated levels were discovered in 3 of them. C was dosed in 10 of the 11 ATTR patients, and it was high in 5 patients (Table 7).

The kidney function was quantified based on the creatinine level by using the MDRD and Cockcroft–Gault formulae and staging the patients accordingly. To that end, the eGFR obtained by the MDRD formula was thought to range between G1 and G4 with most values revolving around G3a and G3b. The Cockcroft–Gault method revealed similar results to the MDRD method. The eGFR range in this case was between stage 1 and stage 4, with most patients being in stage 3 (Table 8).

By comparing the eGFR results obtained by the 2 methods we determined that most of the ATTR patients had stage 3 kidney damage.

For a better correlation between the scintigraphy parameters and renal function we entered the available data in the SPSS statistical analysis program and obtained the following table (Table 9).

### 3.2. AL Patients

The remaining 47 patients showed no cardiac uptake and therefore the ATTR diagnosis was excluded. These patients were thought to have either AL cardiac amyloidosis or some other type of amyloidosis or cardiac pathology. Some of these patients underwent additional investigations in order to determine the exact cause of their heart failure. Following this, only 4 patients were eventually confirmed with AL cardiac amyloidosis by one of FLC dosage, genetic testing or soft tissue biopsy. The other patients were classified as being non-ATTR.

**Figure 5:**
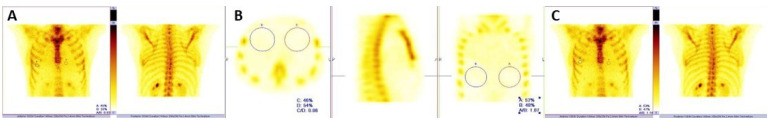
66 year old male patient whose visual score was 0 on the bone scan, H/Rib = 0.83 (image (**A**)), H/Cl region = 0.86 and 1.07 on SPECT images (image (**B**)) followed by 1.14 on static images (image (**C**)); soft tissue biopsy showed the presence of amyloid and FLC dosage determined the presence of kappa lambda chain; eGFR value (calculated by the MDRD and Cockcroft–Gault formula) determined stage 2 kidney disease. Images obtained from the archives of the Nuclear Medicine Laboratory of “Sf. Spiridon” Clinical Emergency Hospital of Iași County.

0 was the Perugini visual score of most AL patients. The score was supported by the ratios for each image type (Figure 5).

However, one patient’s visual score was 1 which could have meant a possible AL cardiac amyloidosis or ATTR in an early stage. The semiquantitative method revealed that the heart uptake was inferior to the rib uptake and compared to the contralateral region it was below the lowest value. Patient numbers from the original lot were maintained to avoid confusion (Table 10).

NT-proBNP, urea and creatinine were dosed for almost all the AL patients. The NT-proBNP results ranged between 3001 and 8000 pg/mL, with most values around 7001 and 8000 pg/mL (Table 11).

Urea was dosed in all 4 patients and just one had elevated levels. Creatinine was dosed in all 4 patients and the levels were normal (Table 12).

Kidney function was also evaluated in the AL patients by using the MDRD and Cockcroft–Gault methods. The MDRD method revealed that all the patients were stage G2. Nevertheless, the Cockcroft–Gault method revealed eGFR between stage 1 and stage 2 (Table 13).

It can be concluded that, for the AL patients, kidney damage does not exceed stage 2 (or G2).

The statistical comparison between AL and ATTR patients in terms of biomarkers dosage and kidney damage showed high NT-proBNP levels in both diseases, however these levels were much higher in some ATTR patients (Table 14).

Urea had similar levels in both conditions. However, as far as kidney damage is concerned, most ATTR patients had higher staging than AL patients (Table 15).

**Figure 6:**
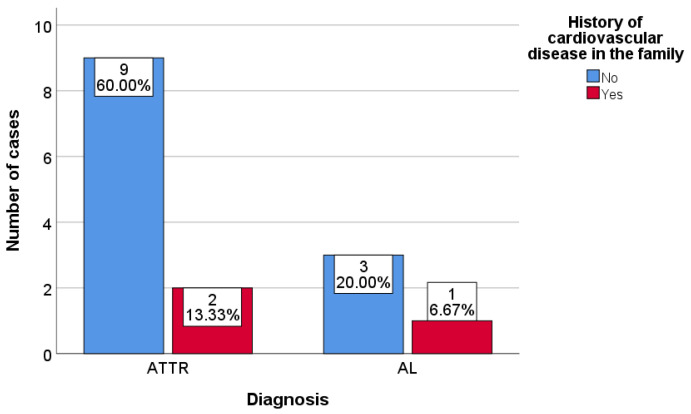
Correlation between the family history of cardiovascular diseases and the bone scan result.

A statistical analysis was also performed based on the family history of heart disease and the bone scan result. The analysis determined that most patients in both ATTR and AL groups were not aware of any kind of cardiovascular disease in the family (Figure 6).

## 4. Discussion

ATTR cardiac amyloidosis is a rare pathology characterized by the deposition of amyloid in the myocardium and coronary arteries determining a thickening of both ventricles, the inter-atrial-ventricular septum and atrioventricular valves. Therefore, diagnosing this rare pathology is a challenge for any practitioner. The gold standard method recommended by the ESC is endomyocardial biopsy. However, this method has a few disadvantages as it is an invasive method, not widely available and cannot distinguish between the subtypes involved. To that end, a correlation between clinical and paraclinical cardiological investigation may be the alternative for a more accurate diagnosis.

According to the ESC, when cardiac amyloidosis is suspected after the clinical and paraclinical examinations the next step is a bone scintigraphy designed to confirm or rule out the presence of ATTR cardiac amyloidosis. The bone scan investigation was based on a personalized protocol compliant with literature and the ASNC and EANM practice points guidelines. Images were acquired early (whole body or static centered-on-the-thorax) and 3 h after the IV injection (whole body, static and SPECT centered-on-the-thorax). Although literature and the practice points do not recommend acquiring early images due to the fact that they have no relevance for the final diagnosis, these images may help in approximating the location and size of the myocardium. After 3 h all the acquired images were processed according to the ASNC and EANM guidelines by using the Perugini visual score and a semiquantitative method in order to obtain a more accurate diagnosis. The semiquantitative method was based on various ROI ratios (heart, rib and contralateral region) made on static and SPECT centered-on-the-thorax. In all the instances the ratio results supported the visual score.

Based on the bone scan results, the patients were classified as ATTR positive (patients with high myocardial uptake of the radiotracer), AL positive (patients with low/absent myocardial uptake of the radiotracer, however, the patients were confirmed with AL by using other investigations) and non-ATTR (patients with low/absent myocardial uptake of the radiotracer, however the patients were not confirmed with AL cardiac amyloidosis). For the ATTR positive patients, another investigation of the patients’ history was made, that was focused especially on cardiovascular diseases. However, most of them could not say if they had a close relative with heart problems. This was supported by the results of the genetic testing that confirmed that some of these patients should have had at least one relative that was suffering from ATTR hereditary cardiac amyloidosis. The statistical analysis showed that most ATTR positive patients (regardless of the subtype) could not specify any kind of cardiac disease that was present in one of their ancestors. To that end, a proper correlation with the patients’ background could not be established. Nonetheless, the patients’ history should not be ignored.

The patients were evaluated according to their bone scan diagnosis and reversed engineering was used to find a correlation between the bone scan and biomarkers. The aim of this correlation is to guide as early as possible the diagnostic algorithm towards a possible cardiac amyloidosis. A more accurate evaluation of these patients required that the biomarkers be selected depending on how many patients underwent the same ones. To that end, the biomarkers most dosed were NT-proBNP, urea and creatinine. In this research, special focus was placed on the ATTR positive patients and on their comparison to the AL positive patients. Due to the small lot of positive patients (ATTR and AL) the risk of bias in our study may be present. The risk factor may require further validation in the future.

NT-proBNP is considered as a heart failure marker. Thus, this biomarker is high whenever there is myocardial distress. The same goes for cardiac amyloidosis. According to literature, this biomarker is high in all heart diseases. Hence, in case of cardiac amyloidosis alone, it cannot offer any kind of new data. Nevertheless, this biomarker and troponine T can be used as markers for evaluating the survival rate. In our case, the ATTR positive patients had NT-proBNP values that were either extreme or not so high compared to the AL patients, most of whom had high NT-proBNP values. Extrapolation may enable us to set a possible range for ATTR and AL patients. Thus, the ATTR patients may have values ranging between 1000–2000 pg/mL or may have extreme values such as over 10,000 pg/mL (a determination which was also supported by the statistical analysis), while the AL patients may have values of 7000–8000 pg/mL.

The urea biomarker was elevated in a few ATTR patients and normal in all the AL patients, therefore it cannot be considered as a possible biomarker in case of cardiac amyloidosis.

Creatinine dosage helped to evaluate kidney function (eGFR). Two formulas were therefore used to approximate the eGFR of the patients: MDRD and Cockcroft-–Gault. According to the MDRD formula, most ATTR patients were stage G3a and G3b compared to the AL patients, who were only G2. The Cockcroft–Gault formula revealed similar results for the ATTR patients (stage 3). However, the AL patients were stage 1 and 2. The differences in the AL patients may be accounted for by the borderline results obtained by the Cockcroft-–Gault formula, which saw some patients classified as stage 1 and not stage 2, despite their borderline results.

By correlating the bone scan results with the biomarkers, it was determined that the current standard biomarkers used to determine the presence of cardiac amyloidosis do not offer an insight for a possible cardiac amyloidosis diagnosis. The standard NT-proBNP biomarker for heart disease is modified regardless of the presence of cardiac amyloidosis (ATTR or AL) or any other type of heart failure. Therefore, relying only on this biomarker may prove insufficient for guiding the physician to the possibility of cardiac amyloidosis.

By definition, amyloidosis is a rare pathology characterized by the deposition of amyloid in different tissues and organ, eventually affecting their normal function. Therefore, amyloid accumulation in the kidney should not be ignored. The paper proved that cardiac amyloidosis has an influence on the eGFR of these patients, which is much more marked in the ATTR patients then in the AL patients, regardless of the method used (MDRD or Cockcroft-Gault). Literature also argues that both ATTRwt and ATTRm can accumulate in the kidney, but they have not been accurately staged yet. Some of the ATTR positive patients underwent genetic testing and thus offered us a possible glimpse for future studies for each ATTR subtype and the staging for kidney disease. Regardless of the mutation presented (Val142Ile or Glu74Gln), patients with ATTRm had a stage G1 or G2 eGFR (Cockroft-Gault: stage 1 and 2). In ATTRwt patients, the eGFR was stage 3 and 4. It is thought that for the hereditary subtype, due to the greater impact on the cardiac muscle, a presentation to the cardiologist may be much quicker compared to ATTRwt patients. To that end, earlier detection of kidney disease may occur much faster and it may have a pivotal role in slowing down its progression. For ATTRwt, due to its unknown etiology and non-hereditary characteristics, cardiac impairment may be detected at a much later time. Therefore, the kidney disease may have progressed greatly before being detected, thus resulting in a higher staging on detection.

Literature shows many cases in which patients with AL cardiac amyloidosis are also diagnosed with kidney disease. Our study revealed stage 2 kidney disease in almost all the AL positive patients. However, the comparison between ATTRm and AL patients revealed that they all had stage 2 kidney disease. Thorough patient background and history investigation may shed some light in such cases.

Reliance on biomarkers such as NT-proBNP, urea and creatinine alone may not substitute the need for a proper medical history, an ECG and an ultrasonography. A decision to refer the patient for a bone scan can be made only by using all the available techniques and data. However, these biomarkers may offer the practitioner an insight into what other organs than the heart may be impaired by cardiac amyloidosis. The patient’s history associated with these biomarkers may suggest the possibility of cardiac amyloidosis in the diagnostic algorithm of any general practitioner. Additional information provided by ECG and ultrasonography would increase the chances of a possible cardiac amyloidosis diagnosis and therefore refer the patient for a bone scintigraphy in order to confirm/rule out the presence of ATTR cardiac amyloidosis. Genetic testing and FLC dosage should always be included in the diagnostic algorithm. Whether these tests are made at the same time with the bone scan (as recommended by the ESC guideline for FLC dosage) or not, they should not be ignored as unnecessary for a more accurate diagnosis.

## 5. Conclusions

Cardiac biomarkers that can determine without further tests the presence of a rare disease such as cardiac amyloidosis would represent a dream come true. However, the current analyzing techniques do not offer a certainty when based only on the read values. Relying on standard cardiological blood tests that include NT-proBNP and troponin T may offer a possible clue that a cardiac disease may be present; nevertheless, it is impossible to set a diagnosis based on these tests alone. This paper tried to determine the ranges between which patients with ATTR and AL may be considered as suffering from cardiac amyloidosis. However, the obtained results were not that conclusive and therefore considering them mainly as predictors of mortality may seem to be the better option. However, cardiac biomarkers associated with eGFR evaluation may prove to be the pivotal point in cardiac amyloidosis diagnostic algorithm. A well investigated background associated with these markers may increase specificity for this rare disease. By completing the analysis with ECG, ultrasonography and bone scan, the chances for detecting and differentiating the cardiac amyloidosis involved increase dramatically. Due to the fact that in the case of cardiac amyloidosis amyloid accumulation may be found in the coronary arteries as well, the presence of these proteins in the renal arteries cannot be ruled out, which may suggest a possible cardio-renal syndrome. The cardio-renal syndrome may also be correlated with the CKD stages. However, these hypotheses will require further research.

## Figures and Tables

**Table 1:** Semiquantitative method classification based on ROI ratios.

Semiquantitative Method		
Ratio	Results	Interpretation
Heart/Rib	>1	Myocardial uptake greater than rib uptake Diagnosis: ATTR cardiac amyloidosis
	<1	Myocardial uptake lower than rib uptake Diagnosis: possible AL cardiac amyloidosis
	1	Myocardial uptake similar to rib uptake Diagnosis: possible ATTR in an early form
Heart/Contralateral region	0–1.49	Absence/slight uptake of the radiopharmaceutical in the myocardium Diagnosis: AL cardiac amyloidosis
	1.5–3	Moderate/increased uptake of the radiopharmaceutical in the myocardium Diagnosis: ATTR cardiac amyloidosis

**Table 2:** Semiquantitative Method, results for ATTR positive patients on static centered-on-the-thorax images.

Semiquantitative Method—Results for ATTR Positive Patients—Static Images			
Patient	Visual Score	Heart to Rib	Heart to Contralateral Side
Patient 4	3	1.68	2.09
Patient 16	3	2.06	2.41
Patient 19	3	2.08	2.14
Patient 20	3	1.85	2.71
Patient 21	3	1.92	2.11
Patient 29	3	1.67	1.6
Patient 45	2	0.91	1.2
Patient 54	2	1.64	1.81
Patient 55	2	1.88	2.07
Patient 56	2	1.18	1.44
Patient 58	3	1.77	2.08

**Table 3:** Semiquantitative Method, results for ATTR positive patients on SPECT centered-on-the-thorax images.

Semiquantitative Method—Results for ATTR Positive Patients—SPECT Images			
Patient	Visual Score	Heart to Contralateral Side	
		Transverse	Coronal
Patient 4	3	2.13	2.5
Patient 16	3	2.97	2.98
Patient 19	3	-	-
Patient 20	3	-	-
Patient 21	3	-	-
Patient 29	3	2.37	0.98
Patient 45	2	-	-
Patient 54	2	2.11	1.79
Patient 55	2	2.8	2.94
Patient 56	2	1.12	1.05
Patient 58	3	2.71	2.25

**Table 4:** Semiquantitative Method, ATTR positive results: comparing ratio results of static images with SPECT images; * heart/rib: >1 = ATTR, <1 = AL, 1 = possible ATTR in an early form; ** heart/contralateral region: 0–1.49 = AL, 1.5–3 = ATTR.

Semiquantitative Method—ATTR Positive Patients					
		Static Centered on the Thorax		SPECT Centered on the Thorax	
Patient	Visual Score	Heart/Rib *	Heart/Contralateral Side **	Heart/Contralateral Side **	
				Transverse	Coronal
Patient 4	3	1.68	2.09	2.13	2.5
Patient 16	3	2.06	2.41	2.97	2.98
Patient 19	3	2.08	2.14	-	-
Patient 20	3	1.85	2.71	-	-
Patient 21	3	1.92	2.11	-	-
Patient 29	3	1.67	1.6	2.37	0.98
Patient 45	2	0.91	1.2	-	-
Patient 54	2	1.64	1.81	2.11	1.79
Patient 55	2	1.88	2.07	2.8	2.94
Patient 56	2	1.18	1.44	1.12	1.05
Patient 58	3	1.77	2.08	2.71	2.25

**Table 5:** Family history of cardiovascular disease for the ATTR positive patients.

Patient	ATTR Subtype	Mutation	Family History of Cardiovascular Disease
Patient 4	ATTRm	Val142Ile	No
Patient 16	ATTRm	Glu74Gln	No
Patient 19	ATTRwt	-	No
Patient 20	ATTRwt	-	No
Patient 21	ATTRm	Glu74Gln	No
Patient 29	-	-	YES
Patient 45	-	-	No
Patient 55	ATTRm	Glu74Gln	No
Patient 56	-	-	YES
Patient 58	-	-	No

**Table 6:** NT-proBNP dosage for ATTR positive patients.

NT-proBNP	0–125	125–1000	1001–2000	2001–3000	3001–4000	4001–5000	5001–6000	6001–7000	7001–8000	8001–9000	9001–10,000	>10,001
Number of patients with ATTR	0	0	3	0	1	0	1	0	0	1	0	2

**Table 7:** Urea and creatinine dosage levels for ATTR patients.

	Urea Normal Value: 15–45 mg/dL	Creatinine Normal Values: 0.6–1.3 mg/dL
Patient 4	44	0.8
Patient 16	25	0.82
Patient 19	-	1.58
Patient 20	-	2.09
Patient 21	35	0.89
Patient 29	103	1.40
Patient 45	-	-
Patient 54	149	1.57
Patient 55	51	0.60
Patient 56	28	0.98
Patient 58	42	1.11

**Table 8:** MDRD and Cockcroft–Gault staging for ATTR positive patients.

	MDRD—Stages						COCKROFT—GAULT—Stages				
Number of patients with ATTR	G1	G2	G3a	G3b	G4	G5	1	2	3	4	5
	2	2	3	2	1	0	2	2	5	1	0

**Table 9:** Parameters correlation between bone scintigraphy parameters and renal function.

	Parameter	Pearson Correlation	*p* Value
ATTR	Urea	−0.036	0.947
	Creatinine	0.503	0.204
	eGFR	−0.185	0.661
	Cl creatinine CG	−0.087	0.838
	Visual score on late acquired images	0.585	0.098
	Ratio Heart/Rib	−0.294	0.443
	Ratio Heart/Contralateral region static centered on the thorax	0.312	0.413
	Ratio Heart/Contralateral region on SPECT images—Transverse incidence	0.401	0.504
	Ratio Heart/Contralateral region on SPECT images—Coronal incidence	0.470	0.530

A *p* < 0.05 is considered statistically significant.

**Table 10:** Semiquantitative Method for AL positive patients.

Semiquantitative Method—AL Positive Patients					
		Static Centered on the Thorax		SPECT Centered on the Thorax	
Patient	Visual Score	Heart to Rib	Heart to Contralateral Side	Heart to Contralateral Side	
				Transverse	Coronal
Patient 1	1	0.91	1.20	1.28	1.06
Patient 10	0	0.66	0.98	-	-
Patient 32	0	1.2	1.67	1.05	1.04
Patient 41	0	0.83	1.14	1.16	0.93

**Table 11:** NT-proBNP dosage for AL positive patients.

NT-proBNP	0–125	125–1000	1001–2000	2001–3000	3001–4000	4001–5000	5001–6000	6001–7000	7001–8000	8001–9000	9001–10,000	>10,001
Number of patients with AL	0	0	0	0	1	0	0	0	2	0	0	0

**Table 12:** Urea dosage levels for AL patients.

	Uree Normal Value: 15–45 mg/dL	Creatinine Normal Values: 0.6–1.3 mg/dL
Patient 1	36	0.82
Patient 10	33	0.93
Patient 32	31	0.74
Patient 41	58	1.06

**Table 13:** MDRD staging for AL positive patients.

	MDRD—Stages						COCKROFT—GAULT—Stages				
	G1	G2	G3a	G3b	G4	G5	1	2	3	4	5
Number of patients with AL	0	4	0	0	0	0	2	2	0	0	0

**Table 14:** Statistical representation of NT-proBNP levels for ATTR and AL positive patients.

Parameter	Total Group (*n* = 59)	AL (*n* = 4)	ATTR (*n* = 11)
NT-proBNP	5796.40 ± 6363.61	6133.33 ± 2651.45	5176.33 ± 3788.09

**Table 15:** Statistical representation of urea and creatinine levels for ATTR and AL positive patients.

Parameter	Total Group (*n* = 59)	AL (*n* = 4)	ATTR (*n* = 11)
Urea	60.34 ± 42.83	39.50 ± 12.50	59.63 ± 43.60
Creatinine	1.32 ± 1.11	0.89 ± 0.14	1.47 ± 1.26
eGFR	85.62 ± 53.01	98.93 ± 8.93	75.43 ± 37.34
Creatinine Clearance	110.10 ± 45.52	139.97 ± 13.23	95.46 ± 46.71
CKD—MDRD			
G1	9 (15.3%)	-	2 (18.2%)
G2	21 (35.6%)	4 (100.0%)	2 (18.2%)
G3a	8 (13.6%)	-	2 (18.2%)
G3b	10 (16.9%)	-	2 (18.2%)
G4	2 (3.4%)	-	1 (9.1%)
G5	2 (3.4%)	-	-
CKD—Cockcroft-Gault formula			
stage 1	14 (23.7%)	2 (50.0%)	2 (18.2%)
stage 2	17 (28.8%)	2 (50.0%)	2 (18.2%)
stage 3	20 (33.9%)	-	5 (45.5%)
stage 4	3 (5.1%)	-	1 (9.1%)

## Data Availability

Available only if requested and anonymously.
